# Case of Chronic Farcy

**Published:** 1850-02

**Authors:** John H. Weir

**Affiliations:** Philadelphia


*Case of Chronic Farcy.* Reported by JOHN H. WEIR, M. D., Philadelphia.

(——) *Novi*, aged 36; was never sick in his youth—of delicate constitution, without bad health; never has had any eruptions on his body, or inflammation of eyes or nose. His parents died of diseases having no relation to the one in question. He has heretofore been a stable boy, and the stable to which he was attached contained 26 horses, eight of which were glandered. He was specially charged with the care of these glandered beasts, and was often obliged to wash their noses and lips, that the disease might not be remarked when out of the stable.

Novi slept in the stable, which was damp and unhealthy; he had done so for three years. He has noticed for some time past that the horses were frequently sick, and from his description, it appears that most of them were affected with chronic farcy. He does not remember at what time exactly he became unwell, nor what was the exact cause of his disease. He ascribes it, however, to a kick on the axilla, which pained him so much as to confine him to bed for a short time. The pain was relieved by bleeding and leeches. He was soon much better, and able to attend to his work.

On the day after commencing his usual work he became suddenly so faint as to be unable to walk, and was soon obliged to seek his bed. This faintness was accompanied with nausea, slight vomiting, but no perceptible fever. Two or three days after he perceived a slight *running from the nose.* He had at the same time considerable pain in the pharynx, with slight difficulty in deglutition. No headach. His treatment at home was confined to simple ptisans and foot baths. A short time after having taken a foot bath he felt a violent pain, both in his arms and legs. The *next* day his legs were covered with tumors, which increased rapidly.

From the commencement of the attack the patient has been confined to bed.

On the 19th August, patient entered Hotel Dieu, service of M. Guenau de Mussy.

At the first examination he presented the following appearance.

General emaciation; great prostration; pulse 70, easily compressed; slight heat of skin; appetite pretty good; tongue red; but little headach. Patient sleeps soundly; perfectly free from any starting in sleep.

On both anterior and posterior pillars of the throat there is much redness. The uvula is of a deep red, swollen, and clotted with whitish *plaques;* the patient is continually *sniffling*, spits rarely, *and always pure saliva.* Nothing remarkable in the anterior portion of the nasal fossæ, which are entirely dry. The excretions are normal and regular. There exists on different parts of the body a large number of fluctuating tumors; one on the *lower part* of the *left thigh*, of the size of a hen's egg; one on the *inner side* of the *right thigh;* one smaller, on the calf of right leg; one on same leg above the last mentioned, and more deeply situated; another below and around the tendo Achillis; one at the inner and inferior portion of the right thigh. The right calf is extremely painful, somewhat reddened, tense and fluctuating, apparently filled with pus. Under the right pectoralis major, precisely at the point where the leeches had been applied, is another and large abscess.

These tumors are painful to the touch, and some of them slightly reddened. There is no appearance of pustules on the body. Patient has never had any severe wounds or cuts; never has taken snuff, and has stopped smoking for several months. There is no pain in the abdomen; no cough. The chest is perfectly healthy, with nothing abnormal about the heart. No abscesses or pustules are to be found in the hair; and no engorgement of the lymphatics can be found in any part of the body. Both feet, the right one particularly, are the seat of a very tense and painful œdema. Motion in the feet and ankles almost destroyed.

℞. Friction of Mercurial Ointment over tumors, with Dover's Powder, grs. xii.

In the evening no change.

20th. Patient slept quietly all night, sweating profusely this morning. The symptoms are generally the same. Pulse 76; tongue dry and red; no expectoration; the tumors are all painful, with the exception of the one over the pectoralis major; the urine is of a dark color, not muddy; there is no deposit on standing, and the odor is natural; there is a perceptible diminution of quantity, and when treated by nitric acid, there is a slight whitish precipitate, which, when heated, leaves no residue.

From the 20th to the 31st the symptoms continued the same; the abscesses, as they formed, were opened, and discharged freely a grayish pus, with the consistence of cream and colored with blood. New abscesses continued to form, and, in consequence, the strength of the patient diminished rapidly. The appetite was gradually lost, and the urine decreasing daily in quantity, becoming, on the 31st, somewhat purulent.

3d September. The urine is yellow, slightly tinged with red, alkaline; no precipitate from nitric acid, and contains a sensible quantity of albumen and salts of lime—but little uric acid.

7th. Patient died this morning without pain, and with mind perfectly clear to the last.

At the autopsy all the internal organs were found healthy, with the exception of the bladder, which showed traces of inflammation, and in some places slight erosion of the mucous coat.

